# Evidence for Menopause as a Sex-Specific Risk Factor for Glaucoma

**DOI:** 10.1007/s10571-021-01179-z

**Published:** 2022-01-04

**Authors:** Amber Douglass, Michael Dattilo, Andrew J. Feola

**Affiliations:** 1 Center for Visual and Neurocognitive Rehabilitation, Atlanta VA Healthcare System, Decatur, GA USA; 2 Department of Ophthalmology, Emory Eye Center, Emory University School of Medicine, B2503, Clinic B Building, 1365B Clifton Road NE, Atlanta, GA 30322 USA; 3 Department of Ophthalmology, Atlanta Veterans Affairs Medical Center, Atlanta, GA USA; 4 Biomedical Engineering, Georgia Institute of Technology and Emory University, Atlanta, GA USA

**Keywords:** Menopause, Glaucoma, Ovariectomy, Biomechanics, Estrogen, Outflow resistance, Retinal ganglion cells, Visual function, Intraocular pressure, Sex specific

## Abstract

Glaucoma is a leading cause of irreversible blindness worldwide and is characterized by progressive loss of visual function and retinal ganglion cells (RGC). Current epidemiological, clinical, and basic science evidence suggest that estrogen plays a role in the aging of the optic nerve. Menopause, a major biological life event affecting all women, coincides with a decrease in circulating sex hormones, such as estrogen. While 59% of the glaucomatous population are females, sex is not considered a risk factor for developing glaucoma. In this review, we explore whether menopause is a sex-specific risk factor for glaucoma. First, we investigate how menopause is defined as a sex-specific risk factor for other pathologies, including cardiovascular disease, osteoarthritis, and bone health. Next, we discuss clinical evidence that highlights the potential role of menopause in glaucoma. We also highlight preclinical studies that demonstrate larger vision and RGC loss following surgical menopause and how estrogen is protective in models of RGC injury. Lastly, we explore how surgical menopause and estrogen signaling are related to risk factors associated with developing glaucoma (e.g., intraocular pressure, aqueous outflow resistance, and ocular biomechanics). We hypothesize that menopause potentially sets the stage to develop glaucoma and therefore is a sex-specific risk factor for this disease.

**Graphical Abstract:**

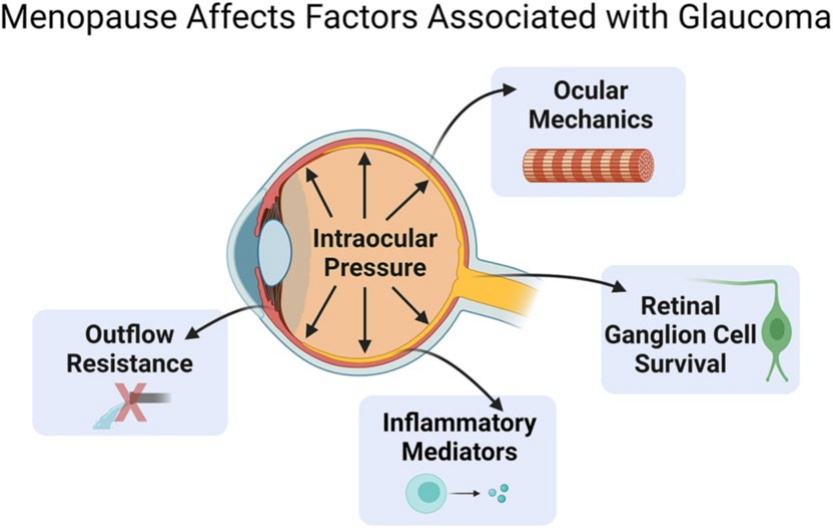

## Introduction

### Overview

Glaucoma is the leading cause of irreversible blindness worldwide and the number of individuals with glaucoma is expected to be over 112 million people by 2040 (Quigley and Broman 2006; Tham et al. 2014). Glaucoma is characterized by a specific pattern of visual field decline and, in advanced stages, remodeling at the optic nerve head (ONH) and lamina cribrosa results in posterior bowing of the lamina cribrosa (Burgoyne 2011; Morrison and Pollack 2003; Campbell et al. 2013). Glaucoma has hallmark structural changes in the eye, including thinning of the retinal nerve fiber layer (RNFL), which is associated with the loss of retinal ganglion cells (RGCs) (Morrison and Pollack 2003). There are roughly 1.2 million RGC axons in humans that relay visual information from the eye to be further processed in the visual cortex (Sterling 1998). Loss of RGCs and their axons are responsible for the visual impairment associated with glaucoma.

While the structural changes and loss of RGCs are common in glaucoma, glaucoma is a group of heterogeneous diseases and not a single disease (Dietze et al. 2021; Allison et al. 2020). Glaucoma is often divided into three broad classifications [open-angle, angle closure (or closed-angle), and developmental] based on the etiology of the disease. The two major forms of glaucoma are open- and closed-angle glaucoma (Dietze et al. 2021; Stein et al. 2021). Primary angle closure glaucoma (PACG) is the major form of glaucoma in Asia and known risk factors, include ethnicity, age, refractive error, sex, and family history (Dietze et al. 2021; Allison et al. 2020; Stein et al. 2021; Zhang et al. 2020; Aryan et al. 2020). Primary open-angle glaucoma (POAG) is the predominant form of glaucoma in the USA, Europe, Africa, and Australia (Morrison and Pollack 2003; Zhang et al. 2021). Known risk factors associated with POAG are elevated intraocular pressure (IOP), age, ethnicity, and family history (Dietze et al. 2021; Allison et al. 2020; Stein et al. 2021; Mahabadi et al. 2021). Although POAG can occur at any level of IOP, ocular hypertension (OHT) remains a major casual risk factor for developing this disease, where OHT is defined as an IOP greater than 21 mmHg. While the mechanisms of open- and closed-angle glaucoma are different, treatment for both involve reducing IOP—as it remains the only modifiable risk factor (Dietze et al. 2021). While lowering IOP is currently the target for treatments, the biomechanical properties of ocular tissues have been proposed as an additional mechanism in the pathophysiology and development of glaucoma (Burgoyne 2011; Campbell et al. 2013; Tamm et al. 2017). The deformation of the ONH due to IOP is largely governed by the mechanical properties (i.e., stiffness) of the posterior eye, especially the sclera (Feola et al. 2016; Feola et al. 2018; Schwaner et al. 2020; Sigal and Ethier 2009). In addition, the cells within the ONH are mechanosensitive and govern the response to the surrounding loads (i.e., the pressure surrounding the ONH) (Burgoyne 2011; Campbell et al. 2013; Tamm et al. 2017).

As modulating IOP is the only currently available treatment for glaucoma, it is necessary to understand the factors that control IOP regulation. In short, IOP is tightly controlled by the production and removal of aqueous humor. Aqueous humor is produced by the ciliary body and provides nutrients and removes waste for the avascular tissues in the anterior chamber (e.g., the cornea and lens) (Morrison and Pollack 2003). Aqueous humor is removed from the eye by the conventional (trabecular meshwork-Schlemm’s canal) and unconventional (uveoscleral) outflow pathways. Outflow resistance is the combined resistance to aqueous humor outflow along the conventional and unconventional outflow pathways (Costagliola et al. 2020); outflow resistance has been shown to increase in POAG (Dietze et al. 2021; Johnson 2006; Moses 1977; Overby et al. 2009; Tamm et al. 2015). In humans, aqueous outflow resistance is mainly controlled through the conventional pathway (Costagliola et al. 2020; Grant 1951), thus the trabecular meshwork (TM) plays a major role in determining IOP (Johnson 2006; Moses 1977; Overby et al. 2009; Tamm et al. 2015). It has been shown that increased outflow resistance correlates with a stiffer TM and that open-angle glaucoma patients have stiffer TMs compared to non-glaucomatous subjects (Overby et al. 2014a, b; Wang et al. 2017a, 2017b).

Although IOP remains an important risk factor for the development and progression of glaucoma, there are certainly other, as of yet unknown, mechanisms that contribute to the development and progression of glaucomatous optic neuropathy. In this review, we explore the potential for menopause as a sex-specific risk factor for developing glaucoma. Based upon our data and the data of other investigators, we propose that menopause and estrogen signaling can influence IOP and aqueous humor outflow resistance, two factors known to be involved in the pathogenesis of glaucoma. We also show that menopause and estrogen signaling can affect cell survival, the cellular response to loading conditions, and ocular biomechanical properties, all of which can also alter a patient’s likelihood of developing glaucoma. We will present data that strongly suggest that menopause and estrogen signaling are sex-specific risk factors for glaucoma development and progression. First, we will explore gender in glaucoma followed by general descriptions of menopause and estrogen signaling. We also examined evidence detailing how menopause is known to affect the biomechanical properties of other organ systems, such as the cardiovascular and musculoskeletal systems, as ocular biomechanical properties are known to play a role in ocular health and the pathophysiology of glaucoma. We will then highlight clinical evidence potentially linking menopause and glaucoma. As the retina and optic nerve are extensions of the central nervous system, we focused our research on the central nervous system to those on menopause and estrogen on the retina and glaucoma. Lastly, we will highlight a growing body of literature that examines surgical menopause (ovariectomy) and estrogen signaling in preclinical models of glaucoma and the effect of surgical menopause and estrogen signaling on factors associated with glaucoma.

### Sex and Glaucoma

Females represent 59% of the glaucoma population or an estimated 41.3 million patients worldwide (Quigley and Broman 2006). In addition, women suffering from glaucoma report a higher rate of visual impairment yet are 24% less likely to seek treatment for glaucoma than men (Quigley and Broman 2006; Vajaranant and Pasquale 2012). These data suggest that glaucoma affects women more severely than men and that the number of affected women remains underreported. Unfortunately, in preclinical research, female animals are frequently omitted, with 75–82% of rodent studies only using male animals (Holdcroft 2007; Hughes 2007, 2019). This makes it nearly impossible to identify underlying causes of a disease or responsiveness to drug treatments that may differ between sexes (Holdcroft 2007; Hughes 2007, 2019). Recent evidence recommends focusing on each sex to determine if there are sex-based differences concerning disease development, progression, and treatment (Holdcroft 2007; Hughes 2007, 2019).

The importance of focusing on issues from a women’s health perspective has been proposed in recent funding policies, including the National Institutes of Health (NIH) and Women’s Veterans Health Research Network (Clayton and Collins 2014; Yano et al. 2010). Thus, in glaucoma the importance of sex has become more relevant. Yet, the effect of sex-specific risk factors in glaucoma is not often considered, likely because sex has not been unequivocally identified as a risk factor for glaucoma (Tielsch et al. 1990; Varma et al. 2004). For example, several studies have shown a higher prevalence of primary open-angle glaucoma (POAG) in males (Kim et al. 2016; Leske et al. 1994; Leibowitz et al. 1980), while others found higher rates in females (Lindblom and Thorburn 1982; Mason et al. 1989; Mitchell et al. 1996; Friedman et al. 2004). Yet other studies failed to find an association between sex and POAG (Hollows and Graham 1966; Klein et al. 1992; Tielsch et al. 1991; Wensor et al. 1998). In primary angle closure glaucoma (PACG), several population studies found that females were more at risk for developing PACG than males, but the underlying reason for a sex difference in PACG prevalence remains unknown (Alsbirk 1976; Congdon et al. 1992; Seah et al. 1997). These studies attempt to specifically identify sex as a risk factor for glaucoma (male vs. females); however, fewer studies have examined if menopause itself is a sex-specific risk factor for developing glaucoma within the female population.

**Fig. 1:**
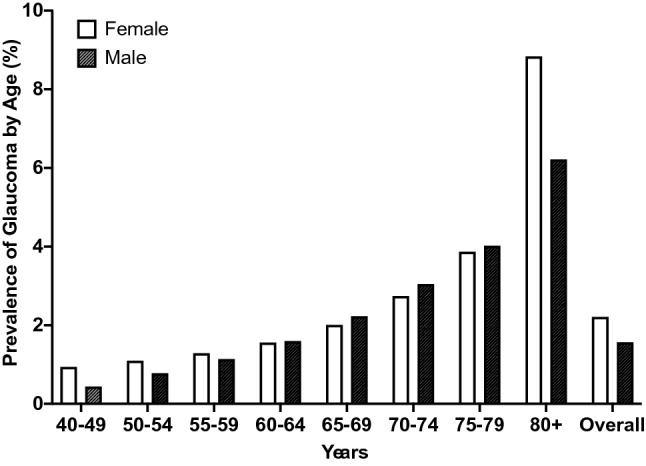
Prevalence of any type of glaucoma by age between males and females by decade. Data were adapted from the NEI database. Overall, females have a higher prevalence of glaucoma compared to males. The relative prevalence of glaucoma in females appears to change throughout life, with a higher prevalence of glaucoma in females occurring in both the early decades (40–59) and later stages of life (80+) compared to males. In comparison, the prevalence of glaucoma in males tends to consistently increase with age

The National Eye Institute (NEI) has recently reported that, in the USA, females have an overall higher prevalence of all types of glaucoma (e.g., POAG and PACG) compared to men (prevalence of 2.21% in females vs. 1.67% in males). The overall higher prevalence of glaucoma in females is often attributed to longer life expectancies in women compared to men (80 vs. 75 years of age) (Agency, C. I. (ed Langley (VA): CIA)(World Factbook [Internet] 2010). This is expected because the likelihood of developing glaucoma is known to increase with age (Morrison and Pollack 2003; Leske et al. 2003; Gordon et al. 2002; Roberts et al. 2018) and women 80+ years of age have a higher prevalence of glaucoma compared to aged men. However, the difference in life expectancy cannot fully explain the overall higher prevalence of all types of glaucoma in females, as middle-aged women (ages 40–59) also have a modestly higher prevalence of glaucoma compared to similarly aged men (Fig. 1). However, this data from the NEI only report the overall prevalence of glaucoma by age without considering gender/sex as a factor associated with developing glaucoma.

The overall differences in the prevalence of glaucoma particularly at younger ages highlight the potential influence of sex-specific risk factors that independently influence the risk of developing glaucoma throughout an individual’s lifetime. Aside from aging, menopause is a major life event unique to females and known to contribute to an increased risk of various pathological conditions in females (Faubion et al. 2015; Mahajan and Patni 2018; Minkin 2019; Monteleone et al. 2018; Nadkar et al. 1999; Nelson 2008; Newson 2018; Takahashi and Johnson 2015). There is a lack of direct evidence to suggest that menopause alone (independent of age) is a risk factor for glaucoma (Klein et al. 1992; Tielsch et al. 1991; Vajaranant et al. 2010). In clinical populations, it is difficult to investigate menopause in isolation, because it is nearly impossible to separate menopause from aging. However, there is indirect evidence suggesting that menopause and estrogens modulate risk factors associated with glaucoma (e.g., IOP) and that the timing of menopause affects the risk of developing glaucoma (Vajaranant and Pasquale 2012; Fotesko et al. 2020).

In this review, we aim to better understand if menopause and estrogen play a role in developing glaucoma. The topic of menopause, estrogen, and glaucoma has recently been discussed (Vajaranant and Pasquale 2012; Fotesko et al. 2020; Wei et al. 2012; Yin and Liu 2008); however, whether menopause or estrogen plays a role in glaucoma development is still debated. Therefore, instead of focusing on sex as a risk for glaucoma we ask the important question: “Is menopause a sex-specific risk factor for glaucoma?” We believe this question remains unsettled and attempt to better understand menopause as a sex-specific risk factor for developing glaucoma. In the following sections, we briefly outline menopause, estrogen signaling, and evidence that menopause is considered a sex-specific risk factor for other pathologies involving the cardiovascular and musculoskeletal systems. Lastly, we will highlight recent literature that has shown how menopause and estrogen play a role related to the development and progression of glaucoma.

### Menopause

We present a short overview of menopause to provide insight into its overall complexity and the importance of the hormonal cycle throughout a woman’s life. This section is not an exhaustive description of the hormonal cycle or menopause, and we encourage readers to read more about the subject (Minkin 2019; Monteleone et al. 2018; Nelson 2008; Takahashi and Johnson 2015).

After puberty, a female has a menstrual cycle that normally occurs once a month and typically occurs for the next 30 years (Allshouse et al. 2018; ACOG Committee Opinion No 2015). The menstrual cycle is regulated by hormones, including those produced by the pituitary gland (luteinizing hormone and follicle-stimulating hormone) which regulate ovulation and ovarian function. The ovaries produce estrogen and progesterone which stimulate the uterus and breasts to prepare the body for fertilization (Mihm et al. 2011). Menopause, which marks the termination of ovarian function, is defined as the absence of menstruation for 12 contiguous months. However, a woman rarely becomes menopausal abruptly; it is common for a woman’s body to begin transitioning through a period called perimenopause.

Perimenopause typically begins in a woman’s 40 s and has several hallmark symptoms (Bacon 2017), including poor sleep, depression, vaginal dryness, dyspareunia, and severely problematic vasomotor symptoms in 33% of women (hot flashes) (Santoro 2016). After perimenopause, women become postmenopausal, often referred to as menopausal, at the average age of 51 years old (Takahashi and Johnson 2015; Santoro 2016; Gold et al. 2013). The age a woman enters menopause is complex, and there are many potential factors, including genetics, reproductive history, lifestyle, early life, and social/environmental influences (Mishra et al. 2019). Women may enter premature menopause, defined as becoming post-menopausal before the age of 40, or early menopause which occurs between the ages of 40 and 45 (Mishra et al. 2019). Overall, data from the InterLACE consortium suggest that 10% of women have premature or early menopause (Mishra et al. 2019). For simplicity, in this review, we will refer to early menopause as entering menopause prior to the age of 45. This equals approximately 390 million women who are expected to enter early menopause based on a global population of ~ 7.8 billion people in 2020. The postmenopausal phase transpires for the remainder of a women’s life and on average accounts for 30–40% of a woman’s total lifespan.

There are many health concerns associated with menopause (Zhu et al. 2018; Asllanaj et al. 2019). Several studies have shown that menopause directly impacts life expectancy and increases a woman’s risk for various diseases, including heart disease, strokes, osteoporosis, and diabetes (Inayat et al. 2017; Anagnostis et al. 2020; Muka et al. 2016; Scarabin 2020; Jiang et al. 2019). Therefore, it is likely that estrogen and progesterone pathways play a key role in women’s health and are often researched as potential treatments after menopause (Allen et al. 2015; Allen et al. 2016; Cutler et al. 2007; Lobo 2017; Thaung Zaw et al. 2018; Hulley and Grady 2004). In this review we will focus on estrogen, which appears to be linked to risks associated with glaucoma (e.g., intraocular pressure (IOP) and aqueous outflow resistance), other ocular pathologies (e.g., AMD and dry eye) (Gibson et al. 2017; Kaarniranta et al. 2015; Patnaik et al. 2020; Truong et al. 2014) and has been suggested as a treatment for glaucoma (Vajaranant and Pasquale 2012; Fotesko et al. 2020; Wei et al. 2012; Yin and Liu 2008; Thaung Zaw et al. 2018; Voogd et al. 2008; Hutchinson et al. 2014).

### Estrogen and Estrogen Signaling

To understand how menopause and estrogen can play a role in women’s health we take a moment to appreciate the complexity of estrogen and estrogen signaling. Estrogen is a major sex hormone throughout the body and while classically considered a female hormone complimentary to testosterone in males, estrogen and testosterone are present and active in both males and females (Ronde et al. 2003; Hammes and Levin 2019).

Estrogen is largely produced in the ovaries in women, but it is also produced in smaller quantities by adipose tissue and locally within various tissues in both sexes (Labrie 2015; Nelson and Bulun 2001). For simplicity, the term “estrogen” is used as an all-encompassing term; however, there are multiple forms of estrogen. The most common forms of estrogen include 17α-estradiol, 17β-estradiol, estriol, and estrone (Hutchinson et al. 2014). It is important to recognize that the prevalence and level of each type of estrogen changes during a woman’s lifetime. Of these forms, 17β-estradiol appears to be the most potent form and is frequently used in animal or basic science studies aimed at hormone replacement therapy. However, the estrogen used in clinical studies often varies in type, source (e.g., synthetic or equine), concentration, and dosage (Yang and Reckelhoff 2011; Chester et al. 2018). This complicates the ability to make direct comparisons between studies and to fully understand the effect of “estrogen,” as any “estrogen” effect or lack of effect may be related to the type, form, or dose of estrogen used in a particular study.

To further confound the role of estrogens, estrogens are known to signal through both endocrine and paracrine mechanisms (Fuentes and Silveyra 2019). There are specific estrogen receptors both on and within cells, including G-coupled Estrogen Receptor (GPER), estrogen receptor-α (ERα), and estrogen receptor-β (ERβ) (Hutchinson et al. 2014; Fuentes and Silveyra 2019; Knowlton and Lee 2012; Baker et al. 2003). The ratio of ERα/β receptors is sometimes important as these two receptors may induce different and sometimes opposing responses from cells. These estrogen receptors are found on cells throughout the body (e.g., neurons and astrocytes) (Aryan et al. 2020; Knowlton and Lee 2012; Kobayashi et al. 1998; Chen et al. 2014; Sniekers et al. 2010; Brennan et al. 2014; Almey et al. 2015) and in the eye these receptors are found in the cornea, retina, lens, and ciliary body (Kobayashi et al. 1998; Ogueta et al. 1999; Suzuki et al. 2001). Lastly, estrogen signaling can occur through genomic and non-genomic mechanisms to activate or suppress gene expression (Fuentes and Silveyra 2019).

Therefore, estrogens are highly diverse in the number of forms, how and where they are produced, and how they can initiate a cellular response. Others have more fully reviewed these complexities (Fuentes and Silveyra 2019; Vrtacnik et al. 2014) and further research is needed to better understand how estrogen signaling is related to developing glaucoma. Estrogen is likely important in ocular health since it is known to affect multiple organ systems, including the central nervous system, it can be produced locally, and ocular tissues, such as the retina and the outflow pathway tissues, have been shown to contain estrogen receptors (Kobayashi et al. 1998; Ogueta et al. 1999; Mabuchi et al. 2010; Youngblood et al. 2020; Wang et al. 2013). This further supports the idea that menopause—a life event that causes a decline of estrogen—would likely play a role in ocular health.

## The Role of Menopause in Developing Pathologies Throughout the Body

### Overview

Below is a review of the literature that provides evidence from clinical and preclinical studies that menopause may be a sex-specific risk factor for developing glaucoma. We attempted to be as inclusive as possible to provide an extensive review of the literature. In brief, we examined public databases from the NIH to determine the prevalence of glaucoma in males and females. We reviewed case studies, clinical studies, and basic science research around the impact of menopause and estrogen on the eye. Our focus was on risk factors associated with developing glaucoma, as well as alterations in cytokine expression, cell survival, biomechanics, and outflow resistance. We examined studies focused on glaucoma, intraocular pressure (or IOP), menopause, hormonal therapy, estrogen, and neuroprotection. We also attempted to include the effects of menopause and estrogen on visual function in multiple injury models in the eye. When examining clinical studies, we cite the original clinical studies or the subsequent studies that performed secondary analysis when relevant.

We start with a literature search of ‘estrogen OR menopause AND eye’ in PubMed results in only 1500 studies. This is a small number of studies considering the numerous studies detailing the effects of menopause and estrogen on the central nervous system (> 24,000 studies); many of these studies focus on mental health and cognitive function (Faubion et al. 2015; Takahashi and Johnson 2015; Albert and Newhouse 2019). Although the eye is considered an extension of the central nervous system, how menopause and estrogen are related to the eye remains unclear and is an area of active research.

**Fig. 2:**
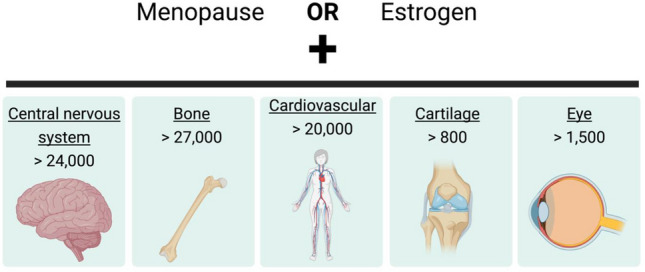
Highlights the vastness of research on menopause or estrogen in different fields of research. These are the results of a PubMed search (performed in May 2021) using menopause or estrogen with a field of study (underlined keyword). The number of publications in each area is listed below and expected to continuously expand as menopause becomes a topic of increased focus

The number of studies focused on menopause and estrogen throughout the body demonstrates its significant role in female health (Fig. 2). For example, there are over 24,000 studies on how menopause and estrogen influence the central nervous system, with many studies focused on mental health and cognitive function (Faubion et al. 2015; Takahashi and Johnson 2015; Albert and Newhouse 2019).

Here, we highlight the cardiovascular and musculoskeletal systems (including cartilage and bone) to demonstrate how menopause modulates the risk for pathologies in these systems. We chose these areas because menopause has been identified as a risk factor for developing musculoskeletal and cardiovascular pathology and menopause has also been shown to influence the biomechanical properties of tissues in each of these organ systems (Feola et al. 2013; Johnston and Ward 2015; Moalli et al. 2008; Urbankova et al. 2018). This is relevant, as ocular biomechanical properties are important for ocular health and are also thought to play a role in developing glaucoma (Burgoyne 2011; Burgoyne et al. 2005). The vastness of this research has improved our understanding of how menopause and estrogen play a role in pathology related to these systems.

### Cardiovascular Disease

The cardiovascular system provides a good example of how menopause, a systemic event, can impact a woman’s risk of developing a disease. Foremost, there are over 20,000 studies on menopause and estrogen in cardiovascular disease (CVD). CVD is one of the leading causes of death in women worldwide (Tandon et al. 2010; Wellons et al. 2012) and is a heterogeneous classification of pathologies, including myocardial infarction, congestive heart failure, hypertension, and stroke. Although CVD is historically considered a major pathology for males, roughly 54% of patients with CVD are female (Garcia et al. 2016) and 70% of females develop CVD after menopause (Pardhe et al. 2017). The age of menopause also modulates the risk of adverse cardiovascular health in women (Muka et al. 2016; Wellons et al. 2012; Zhu et al. 2019a, b). Overall, menopause alone and the timing of menopause impact cardiovascular health in women.

The negative impact of menopause on the cardiovascular system has been extensively detailed in focused reviews (Newson 2018; Khoudary 2017; Zilberman 2018; Shufelt et al. 2018). For example, menopause increases the expression of inflammatory mediators (e.g., TNF, IL-1β, and IL-6) in cardiac myocytes, endothelium cells, and the serum and administering estrogen after menopause lowers their expression (Knowlton and Lee 2012; Cetinkaya Demir et al. 2013; Lin et al. 2018; Rachon 2005; Chung et al. 2009). In addition, menopause and estrogen levels are known to impact the expression and activity of tissue inhibitor of metalloproteinases (TIMPs) and matrix metalloproteinases (MMPs) in the cardiovascular system (Voloshenyuk and Gardner 2010; Mahmoodzadeh et al. 2010; Natoli et al. 2005), which modulate the composition of the extracellular matrix and biomechanical properties of tissues. A recent study using mice found a 50% decrease in cardiac stiffness following surgical menopause compared to control animals (Farre et al. 2018).

### Osteoarthritis

Menopause and estrogen have been heavily investigated in the areas of osteoarthritis (OA) and bone health (> 80,000 studies). OA affects about 27 million people in the USA and females represent 62% of this population (BMUS), B. o. M. D. i. t. U. S. 2020). The development of OA is age related, affecting > 80% of people older than 50 years old (Palo et al. 2015). There is currently no cure or way to reverse the damage caused by OA (Mahajan and Patni 2018); however, estrogen replacement therapies studied on postmenopausal women slowed the progression of and lowered the incidence of OA (Jung et al. 2018; Park et al. 2017; Spector et al. 1997). Still, OA is more prevalent and severe among postmenopausal women (13%) than similarly aged men (10%) (Mahajan and Patni 2018; Sellam and Berenbaum 2013; Srikanth et al. 2005). The rate of OA has been shown to increase at the time of menopause (Tanamas et al. 2011), suggesting that menopause impacts its development and progression (Braidman et al. 2001; Dietrich et al. 2006; Sniekers et al. 2008). Further, studies have shown that estrogen receptors are present in joints (Roman-Blas et al. 2009; Ushiyama et al. 1999) and postmenopausal women with OA have increased inflammatory mediators (IL-1, IL-6, and TNF) in serum and synovial fluids (Sniekers et al. 2008; Liu et al. 2018). In addition, biomechanical properties of the joint and cartilage play a role in OA (Guilak 2011) and the stiffness (aggregate modulus) of articular cartilage decreased after surgical menopause in sheep (Turner et al. 1997).

Similar to the other diseases discussed above, the effect of menopause and estrogen on bone health has been extensively researched (Kanis 1996; Melton et al. 1992; Dobbs et al. 1999). Osteoporosis is a skeletal disease that produces weakened bones and increases the risk of fractures (Beekman et al. 2019). The National Osteoporosis Foundation estimates ten million Americans have osteoporosis and 80% are women. The prevalence of bone loss in females is significantly higher compared to males (16.5% vs 5.1%) (Looker et al. 2017). Early menopause increases the risk for osteoporosis by 1.83 (Francucci et al. 2008; Svejme et al. 2012) and lowers bone mass density by 15% when compared to women with normal menopausal age (Francucci et al. 2008; Francucci et al. 2010). Therefore, there is a direct correlation between the timing of menopause and the development of osteoporosis.

Estrogen plays a major role in bone metabolism in males and females (Khosla et al. 2011, 2012). Menopause leads to bone resorption and poor bone formation. In addition, estrogen levels and osteoclast lifespan are directly proportional (Martin-Millan et al. 2010; McDonald et al. 2021). Indirectly, estrogen deficiency signals cytokines and growth factors, such as IL-1, IL-6, and TNF. These cytokines and growth factors support osteoclast recruitment, differentiation, and survival (Dobbs et al. 1999; Khalid and Krum 2016). With the upregulation in osteoclast activity, the bone becomes brittle, making them more prone to fractures.

## Menopause and Glaucoma Risk

“The Role of Menopause in Developing Pathologies Throughout the Body” section highlighted that menopause is a sex-specific risk factor for multiple basic scientific evidence (e.g., CVD, OA, and bone loss) in different tissues throughout the body. While sex alone is generally not considered a glaucoma risk factor (Tielsch et al. 1990; Varma et al. 2004), we suggest that menopause, similar to the examples in “The Role of Menopause in Developing Pathologies Throughout the Body” section, is a potential sex-specific risk factor for the development of and/or progression of glaucoma. Below we first explore clinical evidence suggesting that menopause has a role in the development of and/or progression of glaucoma. We then explore basic science evidence that menopause is associated with altering inflammatory mediators and cell survival in the retina and with altering biomechanical properties in the eye similar to what has been described in the cardiovascular and musculoskeletal systems (cartilage and bone).

### Epidemiological Studies

It has been difficult to identify if menopause alone is a risk factor for developing glaucoma (Klein et al. 1992; Tielsch et al. 1991; Vajaranant et al. 2010), partly because it is very challenging to distinguish the impacts of aging and menopause in large clinical populations. However, similar to studies on CVD (“Cardiovascular Disease” section), several clinical studies suggest that the age of menopause onset is related to the risk of developing glaucoma (Table 1).

**Table 1:** Comparison between how the timing of menopause influences the risk of CVD and glaucoma in women

% of Women in population	CVD		Glaucoma	
	54% (Garcia et al. 2016)		59% (Quigley and Broman 2006; Vajaranant and Pasquale 2012)	
Stage entering menopause	Directionality of change	Relative risk	Directionality of change	Relative risk
Early menopause	Higher risk	2 (Newson 2018; Wellons et al. 2012; Young and Cho 2019)	Higher risk	2.5–3 (Hulsman et al. 2001; Lam et al. 2014)
Normal menopause	Reference	1	Reference	1
Late menopause	Lower risk	0.88 (Muka et al. 2016; Zhu et al. 2019b)	Lower risk	0.5 (Pasquale et al. 2007)

There are similar trends for how the age of menopause influences the risk of developing CVD and the risk of developing glaucoma (Table 1). This does not appear to be an isolated coincidence as the risk for bone fracture and OA also increases for women who experience early menopause (Mahajan and Patni 2018; Nadkar et al. 1999; Sullivan et al. 2017). As we highlighted above, menopause also influences cytokine expression and biomechanics in each of these organ systems; therefore, similar to the effects of menopause on the health of the cardiovascular and musculoskeletal systems, menopause may be an important factor influencing ocular health. In addition, one study examined the impact of surgical menopause (bilateral oophorectomy) prior to the natural onset of menopause and found that these women were at an increased risk of developing glaucoma later in life compared to similarly aged women who had not undergone surgical menopause (Vajaranant et al. 2014). These results strongly suggest that, at the least, early menopause, either natural or surgical, is associated with an increased risk of glaucoma development.

### Menopause and Intraocular Pressure

**Fig. 3:**
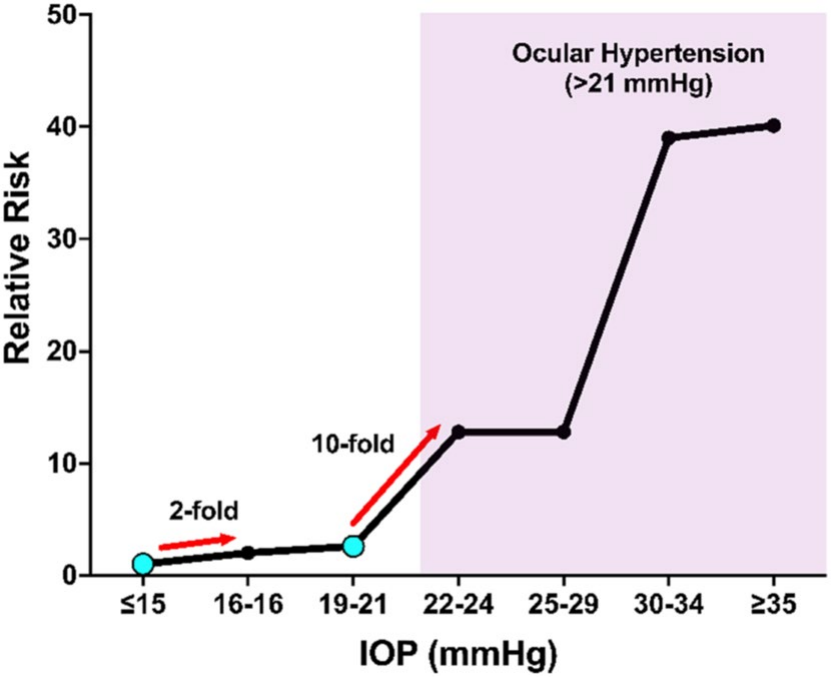
Relative Risk of developing glaucoma as a function of IOP based on data from Sommer et al. (1991) We examine two specific points (cyan dots) where a modest increase in IOP was associated with an increased risk of developing glaucoma. First, an individual with an IOP of 16–18 mmHg has a twofold higher risk of developing glaucoma compared to an individual with an IOP of 15 mmHg or lower. Second, there is a tenfold increased risk of developing glaucoma in an individual with an IOP of 22–24 mmHg compared to an individual with an IOP of 19–21 mmHg. The minor differences in IOP associated with estrogen deficiency/menopause will likely be an important factor to consider when treating women with glaucoma. The shaded region is considered ocular hypertension

IOP, a major causal risk factor for developing glaucoma and currently the only modifiable glaucoma risk factor, is affected by menopause (Vajaranant and Pasquale 2012; Altintas et al. 2004; Vajaranant et al. 2016; Panchami and S. R. et al.. 2013; Kim et al. 2020). Ocular hypertension (OHT), defined as an IOP > 21 mmHg, is associated with an increased risk of developing glaucoma compared to the normotensive population (IOP of 10–21 mmHg) (Morrison and Pollack 2003; Sommer et al. 1991; Sugiyama 2012). Postmenopausal women have a 1.5–3.5 mmHg higher IOP compared to age-matched premenopausal women (Panchami et al. 2013; Qureshi 1996). However, postmenopausal women receiving hormone replacement therapy containing estrogen had a 0.5–3 mmHg lower IOP compared to postmenopausal women not receiving hormone replacement therapy (Vajaranant and Pasquale 2012; Altintas et al. 2004; Vajaranant et al. 2016; Affinito et al. 2003). While these differences appear small, modest increases in IOP have been shown to correlate with an increased risk of developing glaucoma in population-based studies (Fig. 3) (Morrison and Pollack 2003; Sommer et al. 1991). Therefore, these small sustained changes in IOP related to menopause may be another potential factor to consider in women.

### Estrogen Signaling and Glaucoma

There is also evidence suggesting an association between estrogen and estrogen signaling and the development of glaucoma in both sexes. The Rotterdam study found that polymorphisms (haplotype 1) of estrogen receptor-β (ESR2) were associated with an increased risk of open-angle glaucoma in males but not in females, while no haplotypes of estrogen receptor-α (ESR1) altered the risk of developing glaucoma in either sex (Voogd et al. 2008). A separate study by Mabuchi et al. on a Japanese population with normal-tension glaucoma, high-pressure glaucoma, and control patients found that polymorphisms (rs1256031 and rs4986938) in estrogen receptor-β (ESR2) were associated with high-tension glaucoma in females, but not in males (Mabuchi et al. 2010).

In a larger study using the Glaucoma Genes and Environment (GLAUGEN) study and the National Eye Institute Glaucoma Human Genetics Collaboration (NEIGHBOR) consortium examined the association of sex, estrogen metabolism single-nucleotide polymorphisms (SNPs), and primary open-angle glaucoma (POAG) (Pasquale et al. 2013), Pasquale et al. found SNPs along the estrogen metabolic pathway were associated with an increased risk of females developing POAG, but not males (Pasquale et al. 2013). The analysis by Pasquale et al. also found that the catechol-O-methyltransferase gene, which is important for the proper degradation of various substances, including estrogen, dopamine, and epinephrine, showed strong associations with POAG in females.

There are several possible reasons for the different effects of estrogen receptor-β (ESR2) and estrogen metabolism between sexes. First, these studies vary in a few important parameters, such as sample size, populations included, and polymorphism(s), examined. It also highlights evidence that glaucoma is polygenetic, likely because “glaucoma” is a heterogeneous group of diseases and not a single entity. Therefore, many genes likely play a role in the development and progression of glaucoma. A study by Cuellar-Partida et al. found that hereditary factors between sexes may play a role in developing POAG. They also suggest that hormonal signaling may play a factor in its development; however, more work is needed (Cuellar-Partida et al. 2016). Overall, these population studies demonstrate the potential role of estrogen signaling in glaucoma and highlight that additional research is needed to better understand the role of estrogen and menopause in glaucoma.

### Evidence from Basic Science Studies

In addition to the above clinical studies, there are a growing number of preclinical animal studies that demonstrate a potential relationship between menopausal status, estrogen, and glaucoma. Unfortunately, it is impossible to fully replicate the unique reproductive cycle of humans including perimenopause and menopause in preclinical animal models (Diaz Brinton 2012; Koebele and Bimonte-Nelson 2016; Sengupta 2013). Rodents, referring to mice and rats in this review, are a surprisingly good model of hormonal cycles as they naturally have an estrous cycle during their reproductive years. The estrous cycle of mice and rats consists of four phases (called proestrus, estrus, metestrus, and diestrus stages) similar to the menstrual cycle in humans: albeit over a much shorter time frame (4–5 days compared to 28 days) (Ajayi and Akhigbe 2020). Mice and rats also enter estropause between 9 and 12 months of age, which is marked by irregular cycling and hormonal fluctuations. Unlike humans who have a decline in hormones after perimenopause, rodents typically maintain a stable level of estrogen. In rodents, estrogen levels will eventually decline until they reach an anestrus state, but this would require serial direct assessments of hormonal levels to know when each animal reaches the anestrus state (Diaz Brinton 2012; Koebele and Bimonte-Nelson 2016). For more details regarding the advantages and limitations of using a rodent model of aging, hormonal cycles, and menopause, we direct the reader to several reviews (Diaz Brinton 2012; Koebele and Bimonte-Nelson 2016; Sengupta 2013; Kempen et al. 2011). In brief, to model the loss of estrogen in an experimental study, researchers often utilize alternative models of menopause. One of these models relies on performing an ovariectomy (OVX), surgical removal of the ovaries, to initiate ‘menopause’ or low estrogen levels. OVX is a well-established and well-characterized model of menopause (Moalli et al. 2008; Urbankova et al. 2018), known to reliably cause a rapid decline in systemic estrogen and progesterone levels (Koebele and Bimonte-Nelson 2016). OVX is experimentally attractive because it allows induction of a postmenopausal state in animals at a specific time point. This is accepted as an effective animal model to study the consequences of loss of circulating female sex hormones on various conditions, including bone loss, pelvic floor health, osteoarthritis, cardiovascular function, and cognitive function (Feola et al. 2013; Johnston and Ward 2015; Urbankova et al. 2018; Fabricio et al. 2017; Jiang et al. 2015; Li et al. 2014a; Tatchum-Talom et al. 2002; Varbiro et al. 2000; Hoegh-Andersen et al. 2004).

**Fig. 4:**
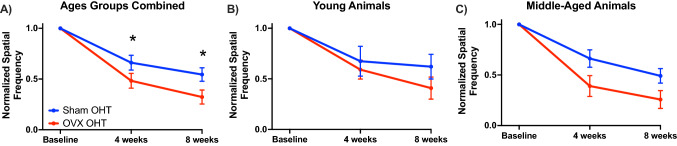
Adopted from Feola et al. (2019) **A** The normalized spatial frequency threshold or visual acuity decreased after ocular hypertension (OHT; ***p < 0.001) in ovariectomized (OVX) and Sham-operated controls (Sham) compared to baseline measurements. Spatial frequency was significantly lower after 4 and 8 weeks in ovariectomized animals after OHT (*p < 0.05; ***p < 0.001) compared to Sham OHT. Data plotted are mean ± S.E.M. **B** Displays the young (age 3–4 months) and C) displays the middle-aged (age 9–10 months) cohorts

Feola et al. examined the impact of age and OVX on visual function and retinal structure in an ocular hypertensive model (OHT) of glaucoma (Feola et al. 2019). They found that OVX in both young (3–4 months old) and middle-aged (9–10 months old) rats resulted in worse visual acuity (spatial frequency threshold) after four and eight weeks of OHT compared to Sham-operated animals (Fig. 4). The spatial frequency threshold is a measure of how well a rodent can see based on its optomotor response, with higher spatial frequencies indicating better visual acuity (Gudapati et al. 2020). These data suggest that OVX at either age heightened vision loss after OHT. OHT also resulted in thinning of the retinal nerve fiber layer (RNFL), but RNFL thinning was not increased with OVX.

**Fig. 5:**
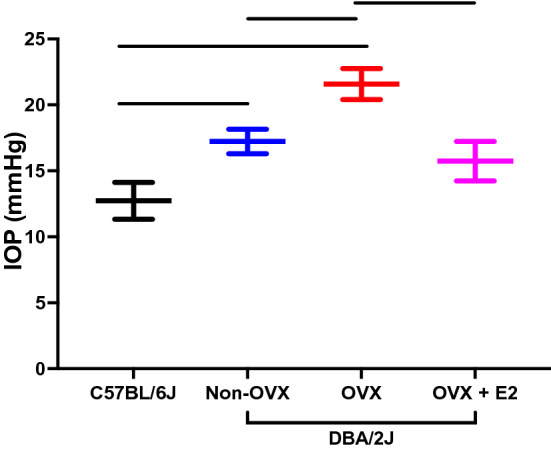
Mice with inherited ocular hypertension (DBA/2J) were divided into non-ovariectomized controls (non-OVX), ovariectomized (OVX), and ovariectomized treated with systemic 17β-estradiol (OVX + E2). Here, C57BL/6J served as a control group. At 6 months, IOP was higher in DBA/2J mice in both non-OVX and OVX animals compared to C57BL/6J mice, with OVX animals having a significantly higher IOP compared to non-OVX animals (*p* < 0.05). Treatment with systemic 17β-estradiol significantly lowered IOP compared to OVX animals (*p* < 0.05). Data were adapted from Zhou et al. (2007) and represented as mean ± SD

Other groups have also examined the impact of OVX and estrogen therapies in experimental models of RGC injury. Prokai-Tatrai et al. found that topical eye drops containing 17β estradiol given after OVX preserved contrast sensitivity (another aspect of visual function) in the same OHT model. Further, they observed that topical estrogen therapy was neuroprotective and preserved RGCs after OHT. Zhou et al. examined the impact of OVX in an inherited model of OHT using DBA/2J mice (Zhou et al. 2007). OVX led to a significant elevation of IOP compared to non-ovariectomized DBA/2 J mice (Fig. 5) (Zhou et al. 2007). The effect of OVX on IOP was ameliorated by systemic estrogen administration; following treatment with systemic estrogen, the IOP was significantly lower compared to the IOP in ovariectomized animals. Zhou et al. also found that OVX was associated with increased RGC loss and with increased expression of inflammatory mediators (IL-18) and mitogen-activated protein kinases (MAPK) in the retina (Zhou et al. 2007). All of these effects of OVX were mitigated after treatment with systemic 17β-estradiol. They further demonstrated that the effects of estrogen were mediated through estrogen receptors by administering an estrogen receptor antagonist (tamoxifen); the protective effect of estrogen was diminished in the groups that received both 17β-estradiol and tamoxifen (Zhou et al. 2007).

Attempting to understand the relationship between estrogen and IOP, Chen et al. generated an aromatase knockout mouse and examined the effect on IOP (Chen et al. 2018). Aromatase, an enzyme that is important to produce estrogen in vivo, was found to only affect IOP in female mice. In aromatase knockout female mice, IOP was increased by nearly 8% in 12-week-old mice and by 20% in 24-week-old mice compared to age-matched wild-type female mice; there was no significant effect on IOP in aromatase knockout male mice compared to age-matched wild-type male mice. This modest elevation in IOP for female mice was associated with a 7–9% decrease in the amount of RGCs in female mice at 24 weeks (Chen et al. 2018).

While these studies involved chronic models of elevated IOP and OVX, a separate study used male rats to examine the neuroprotective effects of estrogen after exposure to an acute elevation in IOP (120 mmHg for 50 min), an ischemic model of RGC damage (Russo et al. 2008). This study found a 28% loss of RGCs after ischemia without treatment, but only a 7% decrease in RGCs in estrogen-treated animals (Russo et al. 2008). However, pretreating a cohort of animals with an estrogen receptor antagonist (ICI 182-780), reduced the efficacy of estrogen in decreasing RGC loss (Russo et al. 2008). These results are similar to how estrogen receptors functioned in RGC preservation in female animals exposed to chronic IOP elevation (Zhou et al. 2007). These data suggest that the neuroprotective effect of estrogen against RGC loss after an injury is partially mediated through estrogen receptors.

**Fig. 6:**
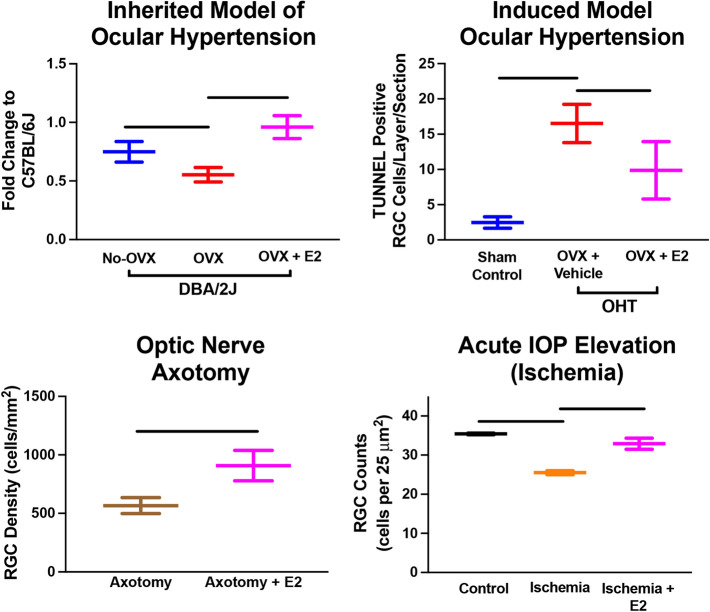
Figures from multiple studies illustrating the neuroprotective effect of 17β-estradiol (E2) in various models of retinal ganglion cell (RGC) injury. These data are all adopted from Prokai-Tatrai et al. (2013), Zhou et al. (2007), Russo et al. (2008), and Nakazawa et al. (2006). All data are presented as mean ± SD. All bars denote significant differences (*p* < 0.05) noted in the literature

In addition to the effect of OVX on visual function and RGCs in a glaucoma model based on IOP, it has also been shown that OVX impacts visual function in a mild optic nerve crush model (Allen et al. 2020). Allen et al. found that OVX in middle-aged (9–10 months old) Long–Evans rats resulted in decreased visual acuity 12 weeks after mild optic nerve crush compared to sham-operated animals (Allen et al. 2020). Similar to ocular hypertensive models of glaucoma, this study showed that menopausal status (OVX) is related to visual function in other models of RGC injury. In addition, a study by Nakazawa et al. examined the impact of OVX on RGCs following optic nerve axotomy using Sprague–Dawley rats (Nakazawa et al. 2006). They found that OVX alone did not change RGC density; however, after axotomy, RGC densities were significantly lower in ovariectomized animals compared to non-ovariectomy animals (Nakazawa et al. 2006). This suggests that OVX alone does not affect RGC density/numbers, but when combined with an insult or stress (e.g., optic nerve axotomy), OVX was associated with increased RGC loss. Nakazawa et al. also showed that a single injection of 17β-estradiol, but not progesterone, was protective against RGC loss. The protective effect of 17β-estradiol was partially mediated through the ERK-c-Fos signaling pathway (Nakazawa et al. 2006). To better characterize the impact of 17β-estradiol treatment, Prokai et al. used mass spectrometry-based proteomics and found 153 up-regulated and 178 down-regulated proteins due to topical treatment in OVX animals (Prokai et al. 2020). Among these were molecules along the MAPK and ERK pathways confirming earlier reports. Overall, they found estrogen treatment-influenced expression along with several physiological processes, including cell signaling, survival, and visual function (Prokai et al. 2020). Together these studies show that estrogen is protective against RGC loss in multiple experimental models of RGC injury (Fig. 6).

**Fig. 7:**
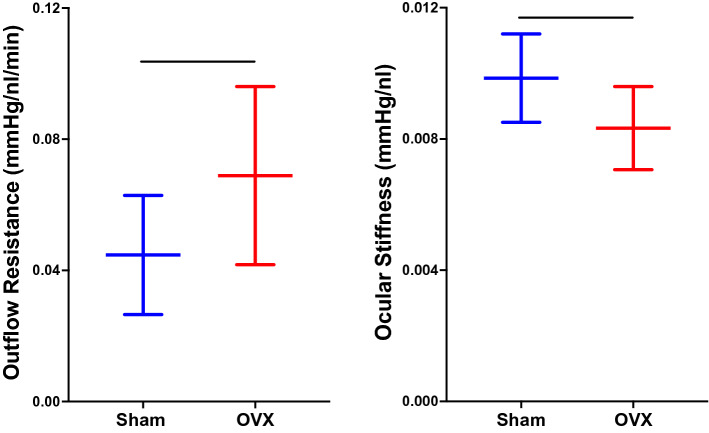
Recent data demonstrating that surgical menopause (ovariectomy; OVX) increases outflow resistance (Left; *p* < 0.05) and decreases ocular stiffness (right; *p* < 0.05). Data are adopted from Feola et al. (2020) and presented as mean ± SD. This highlights that ovariectomy alone is related to factors associated with glaucoma

All of these studies demonstrate that OVX/menopause and estrogen signaling can affect visual function, RGCs, and IOP. In addition to the above studies, several studies have shown that estrogen can affect aqueous humor outflow resistance and biomechanical properties of ocular tissues, two parameters that are known to be affected in some forms of glaucoma (Burgoyne 2011; Campbell et al. 2013; Overby et al. 2009). In particular, several patient studies have shown that aqueous outflow resistance decreases after administration of exogenous estrogen and also during pregnancy (pregnancy typically has elevated levels of systemic estrogen) (Qureshi 1995; Treister and Mannor 1970). In addition to these clinical studies, preclinical studies using female rats found that outflow resistance increased 34% and ocular stiffness decreased nearly 20% after OVX compared to sham-operated animals (Fig. 7) (Feola et al. 2020; Sherwood et al. 2019). The additional effects of OVX on aqueous outflow resistance and ocular biomechanics highlight the effects of menopause on several key physiological factors known to be associated with glaucoma. Although, how OVX impacts ocular biomechanical properties remains unclear, it is known that estrogen modulates biomechanical properties in other tissues, including ligaments, bone, cartilage, cervix, and the vagina (Feola et al. 2013; Moalli et al. 2008; Urbankova et al. 2018; Lin et al. 2018; Komatsuda et al. 2006; Chen et al. 2000), by influencing multiple pathways that modulate cellular responses to mechanical loads (Li et al. 2014b; Richette et al. 2003) and the production of matrix metalloproteinases and collagen (Elliot et al. 2008; Zong et al. 2007; Moalli et al. 2002). The importance of estrogen signaling and its response to mechanical loading has been highlighted by a study examining trabecular meshwork cells subjected to stretch from non-glaucomatous patients (Youngblood et al. 2020). Trabecular meshwork cells play an important role in outflow resistance and these cells differently expressed estrogen receptor 1 after stretch (Youngblood et al. 2020). Therefore, it is likely that the effects of menopause and estrogen signaling on ocular biomechanical properties are mediated through similar, if not the same, pathways.

## Summary

**Fig. 8:**
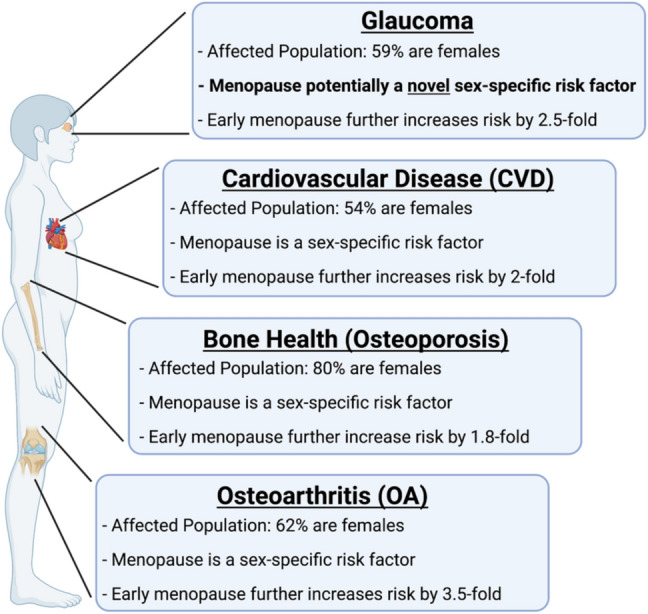
Summary of the pathologies highlighted in this review, proportion of females in the affected population, and the current association with menopause. In cardiovascular disease (CVD), osteoporosis, and osteoarthritis (OA), menopause alone is considered a sex-specific factor and early menopause further increases the risk of developing these pathologies. To date, menopause has not been determined to be associated with developing glaucoma and it is not a consideration when monitoring glaucoma progression or when deciding on treatment. However, we highlight the similarities of glaucoma to these other pathologies and propose that menopause is potentially a novel sex-specific risk factor for developing glaucoma in females

Menopause is well known to be involved as a sex-specific risk factor in the pathogenesis of multiple systemic diseases, such as cardiovascular disease, osteoarthritis, and osteoporosis (Fig. 8). Menopause and estrogen signaling are also known to affect the biomechanical properties of multiple tissues, including the vagina, the cervix, ligaments, bones, and cartilage (Feola et al. 2013; Moalli et al. 2008; Urbankova et al. 2018; Lin et al. 2018; Komatsuda et al. 2006; Chen et al. 2000). Unfortunately, the role of menopause in glaucoma and its effect on ocular biomechanical properties is not as clearly established.

**Fig. 9:**
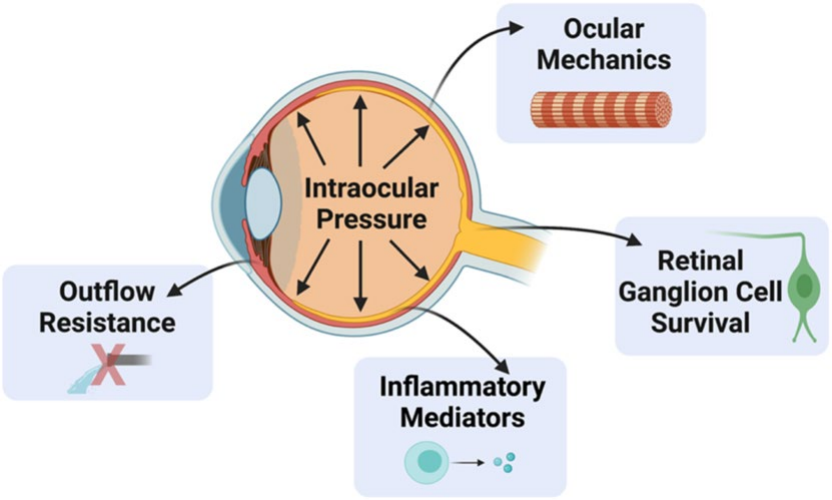
Illustration of how menopausal status and estrogens influence properties throughout the eye. Several of these are direct risk factors for developing glaucoma (e.g., increasing IOP and outflow resistance and decreasing ocular stiffness). However, the role of menopause and estrogen on inflammatory mediators and retinal ganglion cell survival likely plays a key role in long-term ocular health and vision

In this review, we have highlighted multiple clinical and preclinical studies showing that menopause and estrogen signaling influence IOP, RGC survival after injury, aqueous humor outflow resistance, and ocular biomechanics (Fig. 9). Taken together, these studies strongly suggest that menopause and estrogen signaling modulate multiple factors known to be associated with the development of and progression of glaucoma, including IOP, which is a major causal risk factor for developing glaucoma (Fig. 3).

Understanding how menopause influences the development and progression of glaucoma may affect clinical decision-making when evaluating a woman with glaucoma or who is considered a glaucoma suspect. Knowledge of a woman’s reproductive status (i.e., premenopausal, perimenopausal, or postmenopausal) may influence a clinician’s decision about when to initiate IOP lowering therapies and what is an appropriate therapeutic IOP target, given the effects of menopause on IOP. In addition and potentially more important, further knowledge of the effects of menopause and estrogen signaling on glaucoma will likely lead to novel targets for glaucoma treatment. The concept of an estrogen-based treatment for glaucoma has been proposed (Wei et al. 2012; Wu et al. 2020; Dewundara et al. 2016); however, these treatments lack preclinical and clinical evidence to support their widespread use. Based on the literature, glaucoma treatments developed around the impact of menopause and estrogen signaling have the potential to not only influence IOP (similar to all currently available glaucoma treatments) but also to potentially modulate RGC survival, outflow resistance, and ocular biomechanical properties. Therefore, potential therapies aimed to minimize off-target effects of estrogen therapy or exploit specific central nervous system estrogen receptor targets could provide a multi-faceted approach to glaucoma management (Wei et al. 2012; Prokai-Tatrai et al. 2013; Prokai et al. 2015; Prokai-Tatrai and Prokai 2019).

## Conclusion

In conclusion, understanding the impact of menopause on glaucoma and retinal ganglion cell survival has potential clinical applications in the management of glaucoma. Further, as estrogen is known to be neuroprotective, it may potentially have a role in the treatment of non-glaucomatous optic neuropathies, such as ischemic optic neuropathy, compressive optic neuropathy, and traumatic optic neuropathy. The association of menopause and its relationship to glaucoma has an ever-growing body of literature. The similarities of menopause (or ovariectomy) in glaucoma with other major pathologies build a foundation that menopause, a major life event in women, may be a sex-specific risk factor for glaucoma development and/or progression. Further, in ocular research the consistency across laboratories, animal models, and various injury models supports the idea of menopause as a sex-specific risk factor for developing glaucoma that warrants more attention.

## Abbreviations

AMD: Age-related macular degeneration
CVD: Cardiovascular disease
GPER: G-coupled estrogen receptor
ERα: Estrogen receptor alpha
ERβ: Estrogen receptor beta
E2: 17β-Estradiol
IOP: Intraocular pressure
MMP: Matrix metalloproteinases
NEI: National Eye Institute
OA: Osteoarthritis
OHT: Ocular hypertension
ONH: Optic nerve head
OVX: Ovariectomy
PACG: Primary angle closure glaucoma
POAG: Primary open-angle glaucoma
RGC: Retinal ganglion cell
RNFL: Retinal nerve fiber layer
SNP: Single-nucleotide polymorphisms
TIMP: Tissue inhibitor of metalloproteinases
TM: Trabecular meshwork
